# Critical role of G3BP1 in bovine parainfluenza virus type 3 (BPIV3)-inhibition of stress granules formation and viral replication

**DOI:** 10.3389/fimmu.2024.1358036

**Published:** 2024-04-16

**Authors:** Nian Liu, Wei Yang, Lingzhi Luo, Mingshuang Ma, Jin Cui, Xiumei Dong, Yijing Li

**Affiliations:** ^1^ College of Veterinary Medicine, Northeast Agricultural University, Harbin, China; ^2^ Heilongjiang Key Laboratory for Animal Disease Control and Pharmaceutical Development, Harbin, China

**Keywords:** BPIV3, G3BP1, inhibition, stress granules, viral replication

## Abstract

**Background:**

It remains unclear whether BPIV3 infection leads to stress granules formation and whether G3BP1 plays a role in this process and in viral replication. This study aims to clarify the association between BPIV3 and stress granules, explore the effect of G3BP1 on BPIV3 replication, and provide significant insights into the mechanisms by which BPIV3 evades the host’s antiviral immunity to support its own survival.

**Methods:**

Here, we use Immunofluorescence staining to observe the effect of BPIV3 infection on the assembly of stress granules. Meanwhile, the expression changes of eIF2α and G3BP1 were determined. Overexpression or siRNA silencing of intracellular G3BP1 levels was examined for its regulatory control of BPIV3 replication.

**Results:**

We identify that the BPIV3 infection elicited phosphorylation of the eIF2α protein. However, it did not induce the assembly of stress granules; rather, it inhibited the formation of stress granules and downregulated the expression of G3BP1. G3BP1 overexpression facilitated the formation of stress granules within cells and hindered viral replication, while G3BP1 knockdown enhanced BPIV3 expression.

**Conclusion:**

This study suggest that G3BP1 plays a crucial role in BPIV3 suppressing stress granule formation and viral replication.

## 1 Introduction

Bovine parainfluenza is an acute contact infectious disease caused by a bovine parainfluenza virus type 3 (BPIV3) infection. BPIV3 can affect either local or systemic immunity and favor the establishment of secondary bacterial infections, which are common causes of respiratory disease in calves. BPIV3 infection is now a major cause of morbidity and economic losses in cattle worldwide (1–3). The mechanisms underlying the pathogenicity of and immunosuppression by BPIV3 remain unclear, and there is currently no targeted treatment available for BPIV3 infection. A better understanding of the mechanisms underlying BPIV3 pathogenesis and the interactions between BPIV3 and its host will facilitate the development of more effective control strategies.

Various external stress stimuli, such as heat shock, oxidative stress, nutritional deficiencies, and the viral infections can lead to the formation of stress granules (SGs) (4). In response to stress, eukaryotic protein translation is disrupted, eukaryotic translation initiation factor (eIF2α) is phosphorylated, and untranslated mRNAs and untranslated messenger ribonucleoproteins (mRNPs) accumulate in the cytoplasm to form SGs with G3BP1 as the nucleus, thus improving the cell’s ability to respond to external stimuli (5). Viral infection is a powerful external pressure, and host cells need to mobilize multiple pathways to resist viral attacks. SGs can recruit various signal transduction pathway proteins, such as PKR, RIG-I, and MDA-5 to participate in multiple antiviral pathways and play a role in clearing infections (6–8). To evade host antiviral immunity, many viruses have evolved methods to hinder SG assembly. For example, human parainfluenza virus type 3 forms inclusion bodies from auxiliary proteins to segregate newly synthesized viral RNA and inhibit SG formation (9). Middle East respiratory coronavirus auxiliary protein 4a exerts its antagonistic effects by hindering SG formation (10). Short-term infection with porcine epidemic diarrhea virus stimulates the assembly of stress granules, followed by viral activation of caspase 8 that cleaves G3BP1 and depolymerizes SGs (11). The virus targets G3BP1 because G3BP1 is essential for SG formation, and depletion of G3BP1 inhibits SG assembly (12). Typically, SGs are antiviral structures produced by host cells. However, many viruses inhibit or modify SGs to facilitate their replication.

The Ras GTPase-activated (SH3 structural domain) RNA- and protein-binding protein G3BP1 contains several structural domains. These structural domains allow G3BP1 to be actively involved in the regulation of RNA metabolism and multiple signaling pathways, such as regulating mRNA stability and translation, binding to specific transcripts under stress conditions, and activating interferon (IFN)-stimulated gene (ISG) expression (13, 14). G3BP1 has known antiviral functions (15). G3BP1 enhances immune responses against viral invasion by inducing IFN expression and ISG translation. In virus-infected cells, G3BP1 binds to dsRNA produced by viral replication intermediates through the RGG structural domain and enhances RIG-I-induced IFN-β mRNA expression (8); G3BP1, G3BP2, and caprin1 interact to promote ISG translation to synthesize antiviral factors (16). Most importantly, G3BP1 promotes SG assembly to restrict viral protein synthesis. Recent studies have shown that G3BP1 is a central node in protein-protein interaction networks within SGs and that G3BP1 makes an important contribution to SG assembly (12). In addition, G3BP1 interacts with various SG components, including caprin 1 to form a complex that promotes the formation of SGs and USP10 to inhibit the formation of SGs. Even G3BP1 mutants lacking the RGG interaction region can bind caprin 1 and USP10 to affect SG formation (17, 18). However, it remains unclear whether BPIV3 infection leads to SG formation and whether G3BP1 plays a role in this process and in viral replication.

In this study, we found that BPIV3 infection activated phosphorylation of eIF2α but did not induce the SG assembly. It could, however, inhibit the formation of sodium arsenite (SA)-induced SGs at a late stage of infection. Overexpression of G3BP1 inhibited BPIV3 replication, while reducing G3BP1 expression promoted BPIV3 replication, suggesting that G3BP1 plays an important role in BPIV3 infection.

## 2 Materials and methods

### 2.1 Cells and virus

Madin-Darby bovine kidney (MDBK) and HeLa cells were obtained from the American Type Culture Collection, Manassas, USA. All cells were cultured in Dulbecco’s Modified Eagle Medium (DMEM) supplemented with 10% fetal bovine serum (FBS) in a 5% CO_2_ 37°C incubator. BPIV3 (GenBank: HQ530153.1) is a virulent strain that was isolated and adapted to cell culture in the laboratory.

### 2.2 Immunofluorescence antibody assay

MDBK or HeLa cells were inoculated into 96-well plates and cultured overnight at 37°C with 5% CO_2_. Cells were infected with BPIV3 at a multiplicity of infection (MOI) of 2.0. After 1 h of virus adsorption incubation, the medium was replaced with DMEM maintenance medium containing 2% FBS for a period ranging from 3 to 24 h. At different infection time points, cells were fixed using a pre-cooled methanol-acetone mixture, followed by the application of G3BP1 rabbit monoclonal antibody (CST, #45656) at room temperature for 2 h. Subsequently, goat anti-mouse antibody coupled with Alex Fluor 488 antibody and goat anti-rabbit antibody coupled with Alexa Fluor 594 antibody (Proteintech, Rosemont, IL, USA) was applied at room temperature for 1 h. The cells were then incubated for 1 h in 2% DMEM containing 2% FBS (Rosemont). Finally, cell nuclei were stained with DAPI. Cell samples were visualized using a fully automated smart imaging system (BioTek, Vermont, USA).

### 2.3 G3BP1 overexpression plasmid construction

The reference sequence of G3BP1 (GenBank accession number NM_005754.3) was used to design the homologous recombination primers listed in **Table 1** and synthesized by Shanghai Biotech. The G3BP1 gene fragment was amplified from HeLa cells using PCR, and the gene was inserted into the VR-3×FLAG plasmid via homologous recombination with a FLAG-tag at the C-terminus of G3BP1.

**Table 1:** Primer and siRNA sequences.

Name		Sequence (5’-3’)
qPCR primer	G3BP1 Forward primer	GGATGTTATTTGACGCCAACAA
	G3BP1 Reverse primer	TCCCTCTATCGAGTGGAAGACACT
	GAPDH Forward primer	GCATCGGAGGGACTTATGA
	GAPDH Reverse primer	GGGCCATCCACAGTCTTCTG
plasmid construction primer	Forward primer	AGTCACCGTCGTCGAGCCACCATGGTTATGGAGAAGCC
	Reverse primer	CTTTGTAGTCTCTAGGCTGCCTTGGAGCAATGC
siRNA	RNAi#1	AUGAAUCUCCUCAAAGCCUGGTT
	RNAi#2	UACUUUAACAACAUGAGGUGGTT
	RNAi#3	UUUCCCACCACUGUUAAUGCGTT
	RNAi#NC	ACGUGACACGUUCGGAGAATT

### 2.4 Knockdown of G3BP1 by RNA interference

The siRNA reference sequence used for the G3BP1 knockdown (GenBank accession number NM_005754.3), was designed and synthesized by Shanghai Sangon Biotechnology Co. The sequences are listed in **Table 1**. HeLa cells were seeded in 24-well plates in advance and allowed to grow until they reached 50% confluence (approximately 24 h). Using RAN Transfer (Sangong, Shanghai, China), the RNA transfection mixture was prepared according to the ratio indicated in the instruction manual and added to the culture medium after it was left to stand at room temperature for 10 min. The culture medium was replaced 12 h after transfection, and the cells were collected after 48 h of maintenance.

### 2.5 Quantitative real-time PCR analysis

Total RNA from MDBK or HeLa cells was isolated using a Total RNA Extraction Kit (Tengen, China) according to the manufacturer’s instructions. cDNA was synthesized using a Novozymes HiScript III 1st Strand cDNA Synthesis Kit (Novozymes, Beijing, China). quantitative real-time PCR (qRT-PCR) was performed on a Roche LightCycler 480 II with 2× SYBR Green qPCR Master Mix (Selleck, Beijing, China) according to the manufacturer’s instructions. All qRT-PCR experiments were performed in triplicate. Relative gene levels were determined using the $2^{-\Delta \Delta }Ct$ method with GAPDH as an internal control. The primers used for qRT-PCR are listed in **Table 1**.

### 2.6 Western blot analysis

Cellular proteins were extracted using RIPA lysis buffer (Proteintech,Rosemont, IL, USA). Proteins were subjected to sodium dodecyl sulfate-polyacrylamide gel electrophoresis and transferred onto nitrocellulose membranes (Millipore, USA). After blocking with 5% skimmed milk in TBST for 1 h, membranes were incubated with primary antibodies, including phospho-EIF2S1 (Ser51) Polyclonal antibody (Proteintech, Rosemont, IL, USA), EIF2S1 polyclonal antibody (Proteintech, Rosemont, IL, USA), alpha tubulin monoclonal antibody (Proteintech, Rosemont, IL, USA)/G3bp1 (E8N8F) rabbit mAb, then HRP-conjugated affinipure goat anti-mouse IgG or HRP-conjugated affinipure goat anti-rabbit IgG (Proteintech, Rosemont) were labeled for color development. The blots were visualized using an ECL kit.

### 2.7 Assessment of BPIV3 growth in HeLa cells

HeLa cells were inoculated into 24-well plates and cultured overnight at 37°C with 5% CO_2_. Untreated cells were used as a control, and G3BP1 overexpressing cells and G3BP1 knockdown cells were infected with BPIV3 at an MOI of 2.0. Cell cultures were harvested at different time points and inoculated with 10-fold dilutions of the viral solution. Cells were cultured at 37°C with 5% CO_2_ for 3 days. The number of wells and dilutions showing cytopathic effects were recorded, and the TCID_50_ was calculated according to Reed and Muench’s two-component method.

### 2.8 Quantification of SGs

SG formation was determined under a fluorescence microscope, with 3–5 sets of randomly photographed fields of view captured under a high-magnification microscope at 20× magnification, ensuring that the total number of cells was greater than 300. Significant G3BP1 aggregation was used as a marker of SGs. The total number of cells was quantified by counting DAPI-stained nuclei. Cells containing multiple SG marker sites were identified as SG-positive. The percentage of cells containing SGs was calculated as follows: (SG-positive cells/total cells)*100%.

### 2.9 Statistical analysis

Differences between groups of data was assessed using the Student’s t-test, or analysis of variance (ANOVA). Statistical analyses were performed in GraphPad prism software, and results were expressed as the mean ± standard deviation (SD) of at least three independent experiments, with P values < 0.05 considered statistically significant (*P < 0.05; **P < 0.01).

## 3 Results

### 3.1 BPIV3 infection induces phosphorylation of eIF2α but not the formation of SGs

**Figure 1:**
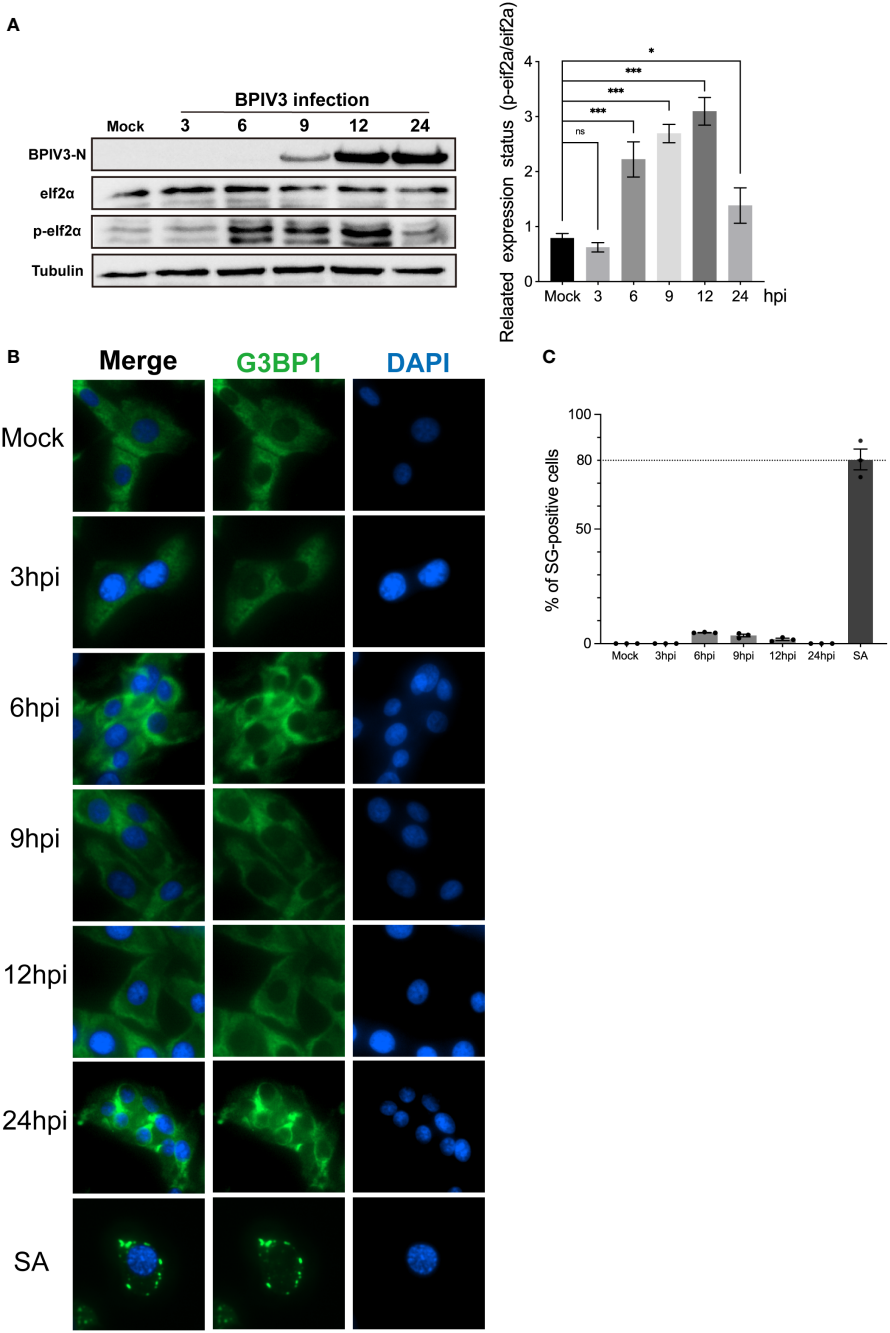
BPIV3 infection induces phosphorylation of eIF2α but not the formation of SGs. **(A)** BPIV3 infection induces eIF2α phosphorylation. MDBK cells were either mock-infected or uninfected with BPIV3 (MOI = 2.0). Cell samples were collected at the indicated time points after infection, and total cellular proteins were extracted and examined for P-eIF2α and eIF2α expression using protein immunoblotting with anti-P-eIF2α, anti-eIF2α, and anti-BPIV3-N antibodies. Tubulin was used as an endogenous control. **(B)** SGs do not form during BPIV3 infections. MDBK cells were inoculated into 96-well plates and cultured for 24 h. AS (0.5 mM) treatment for 45 min served as a positive control, and BPIV3 (MOI = 2.0) infected or uninfected cells were fixed at the indicated time points (3, 6, 9, 12, and 24 h). The cells were detected with rabbit anti-G3BP1 antibody and then probed with goat anti-rabbit-coupled Alexa Fluor 488 antibody. Cell nuclei were restained by DAPI. **(C)** Percentages of cells with SG formation relative to the total number of cells were determined by counting three randomly selected groups in different microscopic fields of view. The calculated percentage represented the proportion of cells with SGs in the total number of cells. Data are expressed as mean ± SD, n = 3. The significance of difference between the groups was performed by ANOVA (* stands for p < 0.05; *** stands for p < 0.001; ns stands for p > 0.05).

Phosphorylation of eIF2α is an initiating condition for SG formation, and regulates SG assembly during viral infection. To determine whether BPIV3 infection induces the formation of SGs, the phosphorylation level of eIF2α was first determined in cells at different infection durations. The experimental results showed that BPIV3 infection led to phosphorylation of eIF2α, and this phosphorylation level was downregulated after 24 h of virus infection (**Figure 1A**). To further investigate the formation of intracellular SGs during BPIV3 infection, MDBK cells were first infected (inoculated with) with BPIV3 (MOI = 2.0), treated with sodium arsenite (SA, 0.5 mM) as a positive control, and the aggregation of G3BP1 in the cells was examined using indirect immunofluorescence. As shown in **Figure 1B**, infection lasted for 3–24 h. There was significant formation of SGs in SA-treated cells, whereas the infected group did not show aggregation of SGs at any time during infection. Further, there was no significant difference in the rates of SG formation between the infected and uninfected groups throughout the BPIV3 infection (**Figure 1C**). The results suggest that BPIV3 infection of host cells stimulates eIF2α phosphorylation but does not induce SG aggregation.

### 3.2 BPIV3 infection inhibits the formation of SGs induced by SA and downregulates G3BP1 protein expression

**Figure 2:**
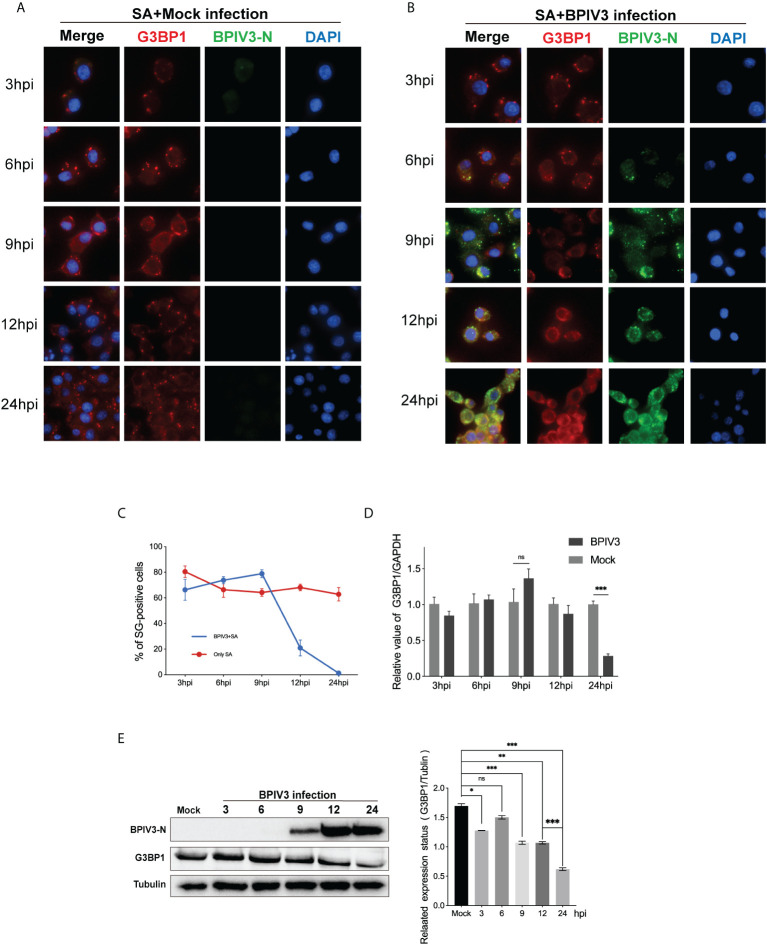
BPIV3 infection inhibits the formation of SGs induced by SA and downregulates G3BP1 protein expression. **(A, B)** Cells were either mock-infected or uninfected with BPIV3 (MOI = 2.0) after treatment with AS (0.5 mM) for 45 min at the indicated infection time points prior to fixation. Cells were stained with rabbit anti-G3BP1 antibody and mouse anti-BPIV3-N antibody, and then labeled with goat anti-rabbit coupled Alexa Fluor 594 antibody and goat anti-mouse coupled Alexa Fluor 488 antibody. Cell nuclei were restained with DAPI. **(C)** BPIV3 infection resulted in a decrease in the percentage of cells forming SGs. The number of cells forming SGs and the total number of cells were counted for AS-treated cells to determine the percentage of cells containing stress granules. **(D)** BPIV3 infection downregulates G3BP1 mRNA levels in MDBK cells. G3BP1 mRNA expression was measured using qRT-PCR at different time points of infection (3, 6, 9, 12, and 24 h) using primers specific for the BPIV3-M gene. GAPDH mRNA expression was used as an internal control to calculate relative levels of gene expression. **(E)** BPIV3 infection downregulates G3BP1 protein levels in MDBK cells. Cell lysates were prepared from the same cell culture samples as those used to perform the qRT-PCR assay. G3BP1 expression was examined using protein blotting with anti-G3BP1 and anti-BPIV3-N antibodies. Tubulin was used as an endogenous control. Data are expressed as mean ± SD, n = 3. The significance of difference between the groups was performed by ANOVA (* stands for p < 0.05; ** stands for p < 0.01; *** stands for p < 0.001; ns stands for p > 0.05).

BPIV3 infection stimulated the phosphorylation of the eIF2α protein but did not induce the formation of SGs, preventing the aggregation and assembly of SG components during viral infection. To determine whether BPIV3 infection inhibited SG assembly, BPIV3-infected or uninfected cells were treated with SA. The rate of SG formation in the uninfected MDBK cell group was consistently > 70% in all cases (**Figure 2A**), which was different from that observed in the BPIV3-infected cells. As BPIV3 viral replication increased, SA-induced SG formation gradually decreased, with almost no SGs formed in the MDBK cells infected for 24 h (**Figure 2B**). Further, the number of the infected cells forming SGs gradually decreased with the duration of viral infection (**Figure 2C**). This suggests that BPIV3 infection hindered the formation of SGs induced by SA.

To determine whether BPIV3 infection affected the SG-nucleating protein G3BP1, intracellular G3BP1 mRNA levels were examined by quantitative reverse transcriptase PCR (qRT-PCR)at differen.t infection times, and protein expression was examined using a G3BP1-specific antibody to label the BPIV3-N protein as an indicator of viral replication. As shown in **Figures 2D, E**, the G3BP1 mRNA transcript level initially increased and then decreased with the progression of viral infection time (**Figure 2D**), while the total amount of protein gradually decreased (**Figure 2E**). This suggested that BPIV3 infection decreased intracellular G3BP1 protein expression and that BPIV3 infection simultaneously inhibited SG assembly and downregulated G3BP1 expression.

### 3.3 Overexpression of G3BP1 enhances SG assembly and inhibits BPIV3 replication

**Figure 3:**
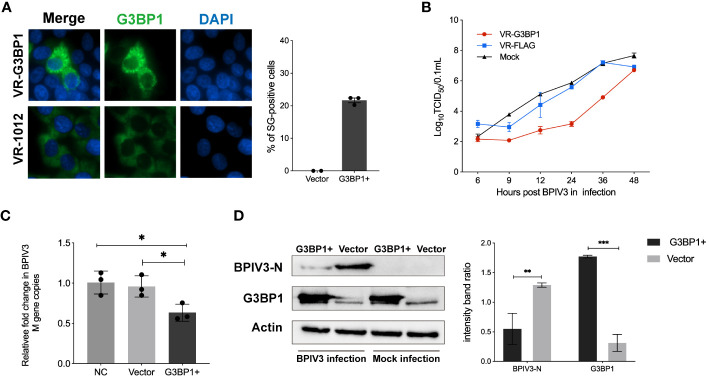
Overexpression of G3BP1 enhances SG assembly and inhibits BPIV3 replication. **(A)** G3BP1 overexpression promotes the formation of SGs in cells. HeLa cells were transfected with VR-G3BP1-3×FLAG or VR-3×FLAG plasmids (100 ng, 96-well plates) for 36 h and detected with rabbit anti-G3BP1 antibody, which was followed by probing with goat anti-rabbit-coupled Alexa Fluor 488 antibody. Cell nuclei were restained with DAPI. The rate of SG formation was calculated after counting the cells with SG formation and the total number of cells. **(B)** Overexpression of G3BP1 inhibits the growth of BPIV3. HeLa cells were transfected with VR-G3BP1-3×FLAG or VR-3×FLAG plasmids; controls were not transfected. After infection with BPIV3 (MOI = 2.0), viral titers in cell culture at different time points were determined using the TCID_50_ method. **(C)** G3BP1 overexpression reduces the BPIV3 viral copy number. HeLa cells were transfected with VR-G3BP1-3×FLAG or VR-3×FLAG plasmid (500 ng, 24-well plate) for 36 h. The controls were not transfected with the plasmid. Simulated infection with BPIV3 (MOI = 2.0) for 24 h was used to detect intracellular viral mRNA levels using qRT-PCR. GAPDH mRNA expression was used as an internal control. **(D)** Overexpression of G3BP1 reduces BPIV3-N protein expression. Cells were infected or not infected with BPIV3 after transfection (MOI = 2.0). Proteins were examined using an anti-G3BP1 antibody against cell lysates and an anti-BPIV3-N antibody. Actin was used as an endogenous cellular reference. Data are expressed as mean ± SD, n = 3. The significance of difference between the groups was performed by ANOVA (* stands for p < 0.05; ** stands for p < 0.01; *** stands for p < 0.001).

SG and its related protein, G3BP1, play an important role in viral replication. Pre-laboratory findings showed that MDBK cells had poor transfection efficiency compared to HeLa cells, whereas BPIV3 could replicate and grow normally in HeLa cells. Therefore, we transfected HeLa cells with VR-G3BP1-3×FLAG to increase the total amount of G3BP1 in the cells and observed whether overexpression of G3BP1 affected BPIV3 replication. Overexpression of G3BP1 stimulated the formation of SGs in HeLa cells, as determined by immunofluorescence (**Figure 3A**). BPIV3-infected G3BP1-overexpressing cells were assessed for viral titers and intracellular viral loads. Viral titers increased more slowly in G3BP1-overexpressing cells than in non-overexpressing G3BP1 cells. At 12, 24, and 36 h after infection, viral titers were more than 10-fold lower in the G3BP1 overexpression group than in the non-overexpression group (**Figure 3B**). The viral load in HeLa cells infected with BPIV3 for 24 h was significantly lower in the G3BP1-overexpression group than in the non-overexpression group (**Figure 3C**). Overexpression of G3BP1 also suppressed the expression of the viral N protein (**Figure 3D**). These data suggest that overexpression of G3BP1 induces the formation of SGs and reduces BPIV3 proliferation efficiency.

### 3.4 Knockdown of G3BP1 promotes BPIV3 replication

**Figure 4:**
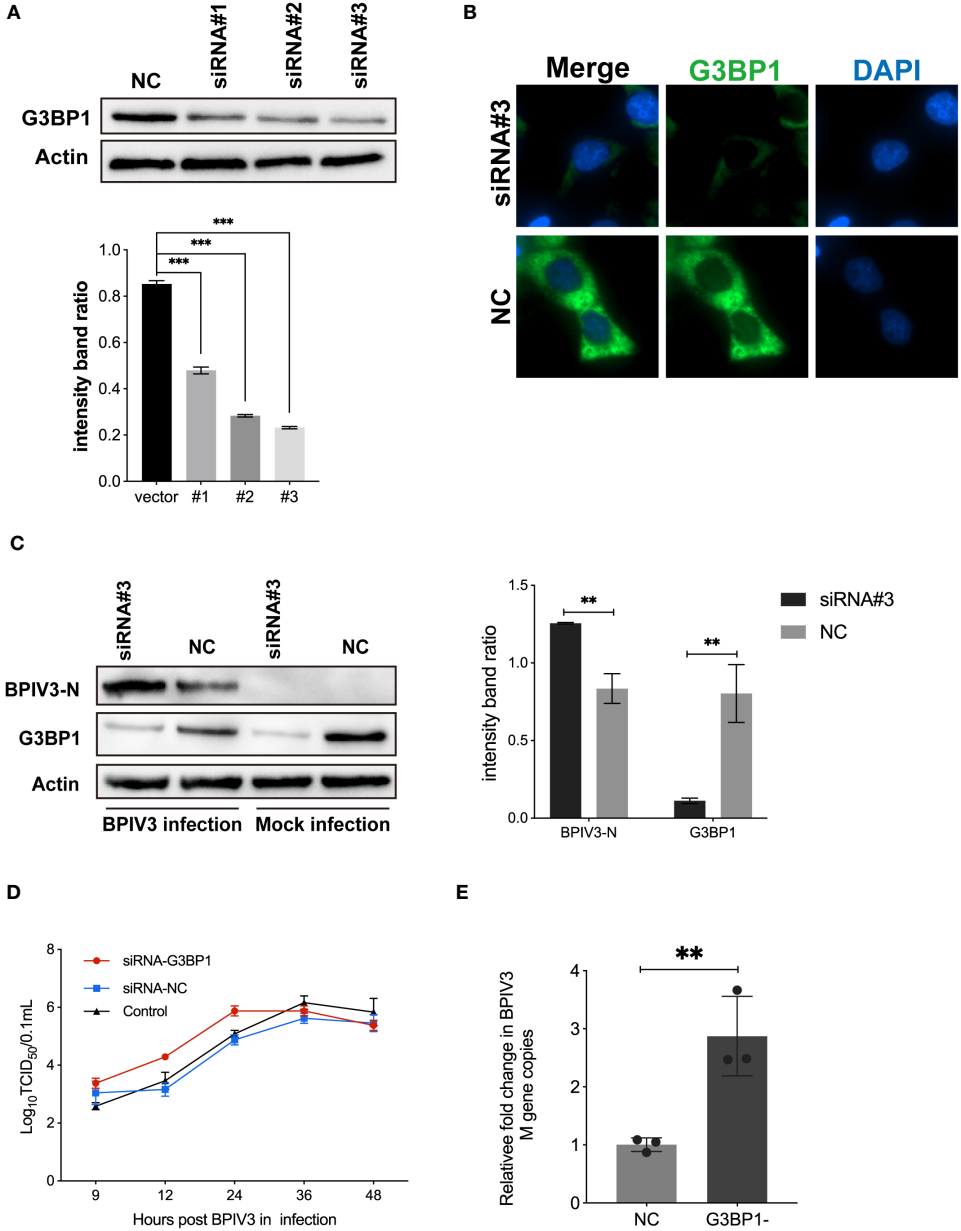
Knockdown of G3BP1 promotes BPIV3 replication. **(A)** siRNA #3 effectively interfered with endogenous expression of G3BP1. HeLa cells were inoculated in 24-well plates for overnight culture and transfected with siRNA #1–3 and siRNA-NC interfering RNA (50 nM), respectively, and cell lysates were prepared 36 h after transfection, and the protein level was determined using an anti-G3BP1 antibody. Actin was used as a reference for endogenous expression. **(B)** HeLa cells were inoculated in 96-well plates and cultured overnight. After 36 h of transfection with siRNA #3 to interfere with G3BP1 expression, the cells were fixed. The cells were detected with rabbit anti-G3BP1 antibody and then probed with goat anti-rabbit coupled Alexa Fluor 488 antibody. Cell nuclei were restained with DAPI. **(C)** Knockdown of G3BP1 resulted in increased BPIV3-N expression. Cells were either infected or not infected with BPIV3 after transfection (MOI = 2.0). After lysing the cells, protein blots were examined using an anti-G3BP1 antibody and an anti-BPIV3-N antibody. Actin was used as an endogenous cellular reference. **(D)** Knockdown of G3BP1 expression promotes growth of BPIV3 titres. HeLa cells were transfected with siRNA #3 or siRNA-NC (50 nM) for 24 h. Controls were not transfected. After infection with BPIV3 (MOI = 2.0), viral titers in cell culture at different time points were determined using the TCID_50_ method. **(E)** Knockdown of G3BP1 increases mRNA levels of BPIV3. HeLa cells were grown to 50% confluence in 24-well plates and transfected with siRNA #3 and siRNA-NC 50 nM, respectively. At 24 h after transfection, mock infection with BPIV3 (MOI = 2.0) was performed for 24 h, and intracellular viral mRNA levels were detected using qRT-PCR. GAPDH mRNA expression was used as an internal control. Data are expressed as mean ± SD, n = 3. The significance of difference between the groups was performed by ANOVA ( ** stands for p < 0.01; *** stands for p < 0.001).

To confirm that G3BP1 plays a role in inhibiting BPIV3 replication, HeLa cells were transfected with three siRNAs. The effects of the constructs on endogenous G3BP1 expression were examined using immunoblotting and immunofluorescence. As shown in **Figures 4A, B**, the RNAi #3 construct effectively reduced endogenous G3BP1 levels compared to the control. This construct was used to knock down G3BP1 expression in HeLa cells in subsequent experiments. Further, G3BP1 knockdown reduced the total amount of intracellular viral N protein in HeLa cells (**Figure 4C**). Subsequently, viral titers and mRNA levels in HeLa cells after knockdown were examined, and an increase in viral titers was accelerated (**Figure 4D**) and the viral load was higher (**Figure 4E**) after knockdown compared to that of the non-knockdown G3BP1 group. These results suggest that a reduction in intracellular G3BP1 promotes BPIV3 replication.

## 4 Discussion

When eukaryotic host organisms are exposed to external stimuli such as oxidative stress and viral infections, specific protein translation “intermediates” are sequestered within the cytoplasm. This phenomenon leads to the development of compact and continuously changing granular formations referred to as SGs. Phosphorylation of eIF2α has been identified as a crucial protein in the signaling pathway for SG formation (19). Phosphorylation of eIF2α, induced by external stimuli, results in inhibition of the translation initiation complex and impedes mRNA translation. Subsequently, core proteins of SGs, including G3BP1, TIAR, and TIA-1, facilitate the formation of messenger ribonucleoprotein complexes (20). After aggregation is complete, mature SGs are formed through protein-protein interactions with multiple proteins involved in signal transduction pathways that are recruited to the core aggregates. Viral infections commonly lead to SG formation. However, our findings indicated that no SGs were formed in the host cells 24 h after infection with BPIV3. Interestingly, our examination of eIF2α phosphorylation in cells that did not form SGs revealed a biphasic pattern, with an initial increase followed by a subsequent decrease as the viral infection progressed. As a crucial mechanism in the formation of SGs, phosphorylation of eIF2α is typically followed by the intracellular assembly of SGs. In the present study, no SGs were identified in cells infected with BPIV3. This suggested that BPIV3 may hinder SG formation, thereby facilitating replication.

To investigate the inhibitory effects of BPIV3 on SG assembly, SG formation was promoted in BPIV3-infected cells using SA. SA is a widely employed potent inducer of SGs, and SA triggers the formation of eIF2α-G3BP1-mediated SGs primarily through activation of the kinase heme-regulated inhibitor, which distinguishes it from the mechanisms employed by most viruses to stimulate SG formation, such as activation of PKR or PERK (21). In this study, we used SA to treat MDBK cells either infected with BPIV3 or not. The rate of SG formation in the cells following SA treatment decreased as the duration of the BPIV3 infection increased. When BPIV3 was cultured onto MDBK cells for 24 h, SA failed to induce SG formation. This suggests that BPIV3 hinders SA-induced SG formation. Furthermore, BPIV3 can impede the aggregation and assembly of SG, similar to the effects of other viral infections. For instance, rotavirus NSP9 protein inhibits SG assembly within host cells during infection (22). Similarly, foot-and-mouth disease virus impedes SG assembly through its leader proteins, which cleave G3BP1 and G3BP2 (23). Additionally, SGs do not form during infection with severe acute respiratory syndrome coronavirus 2 (SARS-CoV-2) because the nuclear capsid protein (NP) of SARS-CoV-2 targets G3BP1 to prevent SG formation (24). SARS-CoV-2 NP interacts with the SG-nucleating protein G3BP1 to inhibit the assembly of SGs. Therefore, it was worth investigating whether BPIV3 targets G3BP1 to inhibit SG formation. During BPIV3 infection, downregulation of G3BP1 transcription and translation was a notable finding. This suggests that BPIV3 significantly impacts the expression of G3BP1. However, further comprehensive investigations are required to elucidate the precise mechanisms by which BPIV3 diminishes G3BP1 expression.

Studies have provided evidence that G3BP1 functions as a pivotal effector molecule, facilitating the formation of SGs and playing a substantial role in antiviral defense mechanisms (25). For instance, the PXXP structural domain of G3BP1 plays a crucial role in facilitating recruitment of the classical antiviral protein PKR to SGs (6). Additionally, it is believed that G3BP1 possesses certain antiviral functions (8). In the present study, overexpression of G3BP1 in HeLa cells facilitated SG formation within host cells. We also observed that G3BP1 inhibited BPIV3 replication. Following overexpression of G3BP1, replication of BPIV3 was diminished, leading to a reduction in viral mRNA levels and viral protein expression. In contrast, G3BP1 downregulation resulted in elevated BPIV3 titers, viral mRNA levels, and viral protein expression levels. Prior research has indicated that G3BP1 can impede replication of porcine epidemic diarrheal virus, SARS-CoV-2, and enteroviruses (6, 26, 27). These previous results align with the outcomes of the current study, suggesting that G3BP1 plays a role in inhibiting viral replication and exerts an antiviral function during BPIV3 infection. A limitation of this study was the use of HeLa cells, which are not natural host cells for BPIV3 infection. This choice was made because of the low transfection efficiency of MDBK cells. Previous studies have also used HeLa cells to investigate bovine-derived viruses (28, 29). The next step in our investigation will involve examining how G3BP1 disrupts BPIV3 replication in host cells.

## 5 Conclusion

In conclusion, the findings of our study indicate that infection with BPIV3 impedes the formation of SGs and can potentially affect the expression of host G3BP1. Overexpression of G3BP1 enhances SG formation in cells, thereby inhibiting replication of BPIV3. Conversely, G3BP1 knockdown promotes BPIV3 replication. Therefore, G3BP1 plays a crucial role in suppressing SG formation induced by BPIV3. These data suggested that BPIV3 infection did not induce the formation of SGs, and that the critical SG regulator, G3BP1, inhibits BPIV3 replication.

## Data availability statement

The original contributions presented in the study are included in the article/supplementary materials, further inquiries can be directed to the corresponding author/s.

## Ethics statement

Ethical approval was not required for the studies on animals in accordance with the local legislation and institutional requirements because only commercially available established cell lines were used.

## Author contributions

NL: Methodology, Writing – original draft. WY: Writing – review & editing, Supervision. LL: Data curation, Writing – review & editing. MM: Formal analysis, Writing – review & editing. JC: Supervision, Writing – review & editing. XD: Conceptualization, Supervision, Writing – review & editing. YL: Supervision, Writing – review & editing.
